# Prevalence of Hospital-Acquired Pneumonia Among Patients With Severe to Critical COVID-19 Pneumonia Given Tocilizumab

**DOI:** 10.7759/cureus.39604

**Published:** 2023-05-28

**Authors:** Lorenzo Abednego B Adre, Josefino S Catangui, Marvin Keith V Bondoc, Kenerham M Abdurahman

**Affiliations:** 1 Department of Medicine, Manila Doctors Hospital, Manila, PHL; 2 Section of Cardiology, Department of Medicine, Manila Doctors Hospital, Manila, PHL; 3 Section of Endocrinology, Diabetes and Metabolism, Chinese General Hospital and Medical Center, Manila, PHL

**Keywords:** coronavirus disease 2019, interleukin-6, nosocomial pneumonia, secondary bacterial infection, interleukin-6 (il-6), hospital-acquired pneumonia, tocilizumab, covid-19

## Abstract

The severe acute respiratory syndrome coronavirus 2 (SARS-CoV-2) pandemic has challenged healthcare systems worldwide since late 2019. The interleukin-6 inhibitor tocilizumab is one of the most studied agents with a proven benefit for patients with severe and critical coronavirus disease 2019 (COVID-19) pneumonia. Known adverse effects of this agent include upper respiratory tract infections, headache, hypertension, and transaminitis. The risk of secondary bacterial complications among patients who were given tocilizumab remains unclear.

A descriptive study was done that included all laboratory-confirmed COVID-19 patients with a severe or critical severity for the year 2021 who received at least one dose of tocilizumab. Of the 1220 laboratory-confirmed COVID-19 patients admitted to Manila Doctors Hospital in the year 2021, a total of 139 patients fulfilled the inclusion criteria and were included in the study. Twenty-one patients, or 15% of the study population, were diagnosed with hospital-acquired pneumonia. This value was similar to previous studies showing the prevalence of secondary bacterial infections among patients who were given tocilizumab. These values could potentially aid clinicians when deciding whether or not to give one or two doses of tocilizumab to patients with severe or critical COVID-19 pneumonia. Given that many patients who are admitted with severe or critical COVID-19 pneumonia often have multiple decompensated comorbidities, the decision to give tocilizumab to manage severe COVID-19 should be weighed against the risk of hospital-acquired pneumonia.

## Introduction

In December 2019, the first cases of coronavirus disease 2019 (COVID-19) were identified, and it has rapidly developed into a global health threat. It has a substantial death toll despite an extensive search for effective treatment regimens. Cytokine release syndrome (CRS) is defined as a systemic inflammatory response that can be triggered by various factors such as infections [1]. CRS may trigger the release of interleukin-6 (IL-6), which plays a major role in acute respiratory distress syndrome (ARDS) and is correlated with poor clinical outcomes.

Further studies have led physicians and scientists alike to enhance the intervention toward CRS. IL-6 inhibitors, specifically tocilizumab, have been one of the most studied agents and are being used in severe and critical COVID-19 pneumonia.

Tocilizumab has been associated with immunosuppression leading to an increased risk of infections such as pneumonia. According to the American Thoracic Society, hospital-acquired pneumonia (HAP) is defined as pneumonia that occurs 48 hours or more after admission, which was not incubating at the time of admission [2].

This study aims to determine the prevalence of HAP among patients with severe to critical COVID-19 pneumonia who have been given at least one dose of tocilizumab during their admission.

This article was presented as an electronic poster at the 53rd Annual Convention of the Philippine College of Physicians on May 8-10, 2023.

Review of related literature

The clinical manifestations of COVID-19 range from an asymptomatic carrier, mild acute respiratory disease, and severe pneumonia, to ARDS [3]. The overall fatality rate was 6.36% [4], and most of the deaths were attributed to severe cases of COVID-19 [5].

The COVID-19 severity classification was defined by the Philippine COVID-19 Living Recommendations. Mild COVID-19 was defined as cases with no pneumonia or desaturation, acute onset of fever and cough, or any three or more of the following: fever, cough, coryza, sore throat, diarrhea, anorexia/nausea/vomiting, loss of sense of smell or taste, general weakness/body malaise/fatigue, headache, and myalgia. Moderate COVID-19 was defined as cases with pneumonia but no difficulty of breathing or shortness of breath, a respiratory rate of less than 30 cycles per minute, oxygen saturation greater than or equal to 94% at room air, or cases without pneumonia but with risk factors for progression. These risk factors include advanced age (>60 years) or comorbidities. Severe COVID-19 was defined as pneumonia and any of the following: signs of respiratory distress, oxygen saturation less than 94% at room air, a respiratory rate greater than 30 cycles per minute, or the need for oxygen support. Critical COVID-19 was defined as pneumonia and any one of the following: impending respiratory failure requiring high-flow oxygen, non-invasive or invasive ventilation, ARDS, sepsis or shock, deteriorating sensorium, multi-organ failure, or thrombosis. Pneumonia in the context of COVID-19 is defined as evidence of lower respiratory disease during clinical assessment (e.g. cough, fever, or crackles on auscultation) and/or imaging (plain chest films, ultrasound, and CT scan).

COVID-19 is associated with complicated pathogenesis; severe SARS-CoV-2 infections suggest that CRS plays a crucial pathogenic role [6-8]. Many proinflammatory cytokines are involved in the CRS, and IL-6 is the most important [9]. Excessive systemic inflammation and elevated IL-6 levels resulting from dysregulated host immune responses [10] are associated with adverse clinical outcomes in patients hospitalized with COVID-19. Anti-IL-6 agents have been proposed as one of the promising treatment regimens for COVID-19 [11]. This led to the beginning of several randomized clinical trials conducted to assess the efficacy of IL-6 antagonists in patients with COVID-19. The IL-6 antagonists commonly investigated were monoclonal antibodies that bind either to membrane-bound and soluble IL-6 receptors such as tocilizumab and sarilumab or directly to IL-6 such as siltuximab.

Tocilizumab is a humanized monoclonal inhibitor of the pro-inflammatory cytokine IL-6 and is licensed for use in the clinical management of CRS. Tocilizumab was approved by the FDA in 2017 for treating CRS [12]. A study by Alattar et al. showed that tocilizumab could reduce aberrant immune response-driven pulmonary manifestations of severe COVID-19 [13]. It can target both membrane-bound and soluble forms of the IL-6 receptor, and several studies have evaluated its efficacy for treating severe COVID-19. According to the Philippine COVID-19 Living Recommendations, tocilizumab is indicated in patients showing signs of rapid respiratory deterioration or patients requiring high doses of oxygen (high-flow nasal cannula, noninvasive or invasive mechanical ventilation) with elevated biomarkers of inflammation such as C-reactive protein. For each additional day of delay from the admission to tocilizumab administration, the odds of receiving mechanical ventilation independently increase by 21% (95% CI: 1.08, 1.38, p = 0.002) [14].

The use of immune-based therapies comes with certain risks. It is a double-edged sword that if not judiciously used, could pose more harm than cure [15]. The main concern regarding tocilizumab therapy is the occurrence of severe infections [16]. Among its well-described adverse events are upper respiratory tract infections, headache, hypertension, and alanine aminotransferase (ALT) rise [17]. Clinical studies of patients with COVID-19 have evoked some safety concerns. The risk of secondary infection complications remains unclear. Tocilizumab may be associated with opportunistic infections [17]. Patients who received one dose of tocilizumab had secondary infections such as candidemia and pulmonary aspergillosis [18].

Among the studies with a comparison group, six studies found a higher rate of infections among treated, compared with untreated [19]. The rate of infection was lower in patients treated with a single dose compared with patients treated with two doses of tocilizumab.

A study by Guaraldi et al. showed that 24 (13%) of 179 patients treated with tocilizumab were diagnosed with new infections, which included bloodstream infections, bacterial pneumonia, candidemia, urinary tract infection, *Pneumocystis jirovecii* pneumonia, invasive aspergillosis, hepatitis B virus reactivation, and herpes simplex virus 1 reactivation [20].

Currently, there is no local study available showing the association of tocilizumab with the development of severe HAP in COVID-19 patients. A study by Aborque et al. from the University of Baguio showed that the clinical outcome of the COVID-19 patients treated with immunomodulators was as follows: 85.09% were discharged in improved condition while a 24.1% mortality rate was noted among those who received both dexamethasone and tocilizumab [15]. One ongoing study is being conducted at the University of the Philippines Manila, entitled "Intravenous Tocilizumab in the Treatment of Patients With Severe and Critical COVID-19-Related Pneumonia"; however, the prevalence of HAP is not included in their primary and secondary outcomes.

## Materials and methods

Research site and plan

The charts of all patients who had a laboratory-confirmed COVID-19 infection and were admitted from January to December 2021 were retrieved from the records section of Manila Doctors Hospital, a private tertiary-level hospital. Each chart was then reviewed to determine which patients would be included in the study based on the inclusion and exclusion criteria.

Population

A list of all patients with a positive COVID-19 nasopharyngeal reverse transcription-polymerase chain reaction (RT-PCR) or rapid antigen test who were admitted to Manila Doctors Hospital from January to December 2021 was retrieved. The charts of these patients were then reviewed to determine which of the patients diagnosed with severe or critical COVID-19 pneumonia who were given at least one dose of tocilizumab developed HAP during their admission based on the 2016 Infectious Diseases Society of America (IDSA)/American Thoracic Society (ATS) guidelines [21]. The diagnosis is based on the presence of a new lung infiltrate on imaging and clinical evidence suggestive of HAP (fever, purulent sputum, desaturations, and leukocytosis) that are noted at least 48 hours after admission and are not thought to be incubating at the time of admission. In addition to this, only patients with a positive growth on blood, sputum, or endotracheal aspirate were considered to have a diagnosis of HAP.

All of the patients included in the study also received 6 mg of intravenous dexamethasone for 10 days and intravenous remdesivir for five to 10 days.

Inclusion and exclusion criteria

Patients aged 18-60 years with no previous vaccinations for COVID-19 and who received at least one dose of tocilizumab during admission were included in the study. Unvaccinated patients aged 18-60 years diagnosed with laboratory-confirmed severe or critical COVID-19 pneumonia on admission using either SARS-CoV-2 RT-PCR or COVID-19 rapid antigen test, who received at least one dose of tocilizumab during admission were included in the study. Tocilizumab was given as an IV infusion of 400 mg diluted in enough plain saline solution for a total of 250 mL to run for two hours.

Patients younger than 18 years or older than 60 years, those who had a previous COVID-19 infection, those who received any type of vaccination for COVID-19 as well as those with known causes of primary (e.g. B-cell immunodeficiencies, T-cell immunodeficiencies, severe combined immunodeficiency (SCID), complement defects, and phagocyte disorders) or secondary immunodeficiencies (e.g. protein-calorie malnutrition, history of organ transplants, solid or hematologic malignancies, history of immunosuppressive medications prior to admission, HIV, and systemic lupus erythematosus) were not included in the study.

Methodology

A list of all patients diagnosed with severe to critical COVID-19 pneumonia who were admitted to Manila Doctors Hospital from January to December 2021 was compiled. The charts of these patients were then reviewed to determine which of them developed HAP during their admission based on the 2016 IDSA/ATS guidelines [21]. The clinical findings and imaging of all patients diagnosed with HAP were reviewed by a consultant pulmonologist.

Data collection plan

The baseline arterial blood gas (ABG) and inflammatory markers (ferritin, high-sensitivity C-reactive protein, D-dimer, and procalcitonin) along with the official interpretation of the first plain chest film provided by the Department of Radiology were collected for all patients included in the study. This provided the basis for the initial classification of the severity of COVID-19 pneumonia.

For patients who were diagnosed with HAP during their admission, the patient's vital signs at the time of diagnosis were reviewed along with the official interpretation of plain chest films done at least 48 hours after admission, as well as any positive blood or sputum culture results where the sample was sent at least 48 hours after admission.

To be able to compare the data with those of other similar studies, baseline characteristics such as age, sex, body mass index (BMI), and comorbidities were included.

Statistical analysis

An in-house hospital statistician was consulted for this study. STATA 13.1 (StataCorp LLC, College Station, TX) [22] was used for statistical analysis. Descriptive statistics were used to summarize the demographic and clinical characteristics of the patients. Frequency and proportion were used for categorical variables, median and interquartile values for non-normally distributed continuous variables, and mean and standard deviation for normally distributed continuous variables.

The 95% confidence interval was determined using binary logistic regression to check for possible correlation between the characteristics of the development of HAP. All statistical tests used were two-tailed tests. The Shapiro-Wilk test was used to test the normality of the continuous variables. Missing values were neither replaced nor estimated. Null hypotheses will be rejected at 0.05 α-level of significance.

## Results

Table 1 shows the baseline characteristics of patients with and without HAP. A total of 1220 laboratory-confirmed COVID-19 patients were admitted to Manila Doctors Hospital in the year 2021. A total of 139 patients fulfilled the inclusion criteria and were included in the study. The mean age of this population was 49 years. A total of 98 patients were male, and 41 were female. Hypertension, diabetes mellitus, and dyslipidemia were the three most common comorbidities in this population, accounting for 90.64% of all the comorbidities in the patient population. Most of the patients were either obese or overweight. Only 15.11% of the patients had a normal BMI, and none of them were classified as underweight. The mean BMI among patients who were diagnosed with HAP was 30.14 ± 8.11 and 27.52 ± 4.74 among those who were not diagnosed with HAP. There is a statistically significant difference between the mean BMI of patients with and without HAP.

**Table 1:** Baseline characteristics of patients with and without hospital-acquired pneumonia IHD: ischemic heart disease; CKD: chronic kidney disease; SD: standard deviation; BMI: body mass index.

	Total	Hospital-acquired pneumonia		P-value
	(n = 139)	With	Without	
		(n = 21, 15%)	(n = 118, 85%)	
	Mean ± SD			
Age (in years)	48.63 ± 9.29	51.33 ± 7.07	48.14 ± 9.58	0.148
Sex				0.122
Male	98	18	80	
Female	41	3	38	
Height (cm)	164.47 ± 7.90	163.38 ± 6.47	164.67 ± 8.14	0.495
Weight (kg)	75.6 ± 16.39	80.76 ± 25.2	74.69 ± 14.24	0.118
BMI	27.92 ± 5.43	30.14 ± 8.11	27.52 ± 4.74	0.041
Underweight	0	0	0	0.263
Normal	21	2	19	
Overweight	21	1	20	
Obese	97	18	79	
Comorbidities				
Hypertension	68	13	55	0.24
Diabetes mellitus	46	10	36	0.137
Dyslipidemia	12	2	10	1
IHD	7	1	6	1
Asthma	7	1	6	1
CKD	4	1	3	0.485
Hyperuricemia	3	2	1	0.06
Others	12	2	10	1

Of the 139 severe and critical COVID-19 patients who were given tocilizumab and included in the study, 21 (15%) were diagnosed with bacterial HAP based on the 2016 IDA/ATS guidelines and were all positive for an organism in blood, endotracheal aspirate, or sputum culture studies.

Table 2 shows a comparison of the median and interquartile values of the inflammatory markers for patients with and without HAP. The median ferritin among patients diagnosed with HAP was 2000 mcg/L (1525-2392) and 1477 mcg/L (828-2111.1) among patients who were not diagnosed with HAP. The median D-dimer among patients with HAP was 1822 ng/mL (923-10000) and 803 ng/mL (506-1608) among patients without HAP. The median partial pressure of oxygen (PaO​​​​​_2_)/fraction of inspired oxygen (FiO_2_) ratio was 133 (73.75-225.3) among patients diagnosed with HAP and 211.11 (131-302.85) among patients who were not diagnosed with HAP. The median high-sensitivity C-reactive protein (hs-CRP) value was 86.72 mg/dL (43.9-144) among patients with HAP and 86.75 mg/dL (43-146) among patients without HAP.

**Table 2:** Median (interquartile range) of inflammatory marker values for patients with and without hospital-acquired pneumonia Hs-CRP: high-sensitivity C-reactive protein; PaO2: partial pressure of oxygen; FiO2: fraction of inspired oxygen.

	Total	Hospital-acquired pneumonia		P-value
	(n = 139)	With	Without	
		(n = 21, 15%)	(n = 118, 85%)	
	Median (interquartile range)			
Inflammatory markers				
Ferritin (mcg/L)	1605	2000	1477	0.048
	(847 to 2220)	(1525 to 2392)	(828 to 2111.1)	
Hs-CRP (mg/dL)	86.72	86.72	86.75	0.853
	(43 to 146)	(43.9 to 144)	(43 to 146)	
D-dimer (ng/mL D-DU)	876	1822	803	0.002
	(616 to 1879)	(923 to 10000)	(506 to 1608)	
Procalcitonin (ng/mL)	0.15	0.26	0.15	0.1
	(0.05 to 0.43)	(0.14 to 0.72)	(0.05 to 0.32)	
PaO2/FiO2	197.91	133	211.11	0.043
	(122 to 299.5)	(73.75 to 225.3)	(131 to 302.85)	

There was a statistically significant difference between the median ferritin, D-dimer, and PaO​​​​​​_2_/FiO_2_ values of patients with and without pneumonia.

Table 3 shows a breakdown of the patient population based on the inflammatory marker value cutoffs used in Manila Doctors Hospital. The ferritin, D-dimer, and hs-CRP values of most of the patients included in the study are considered high risk while the procalcitonin values of most of the patients are considered low risk.

**Table 3:** Inflammatory marker risk stratification based on hospital laboratory value cutoffs Hs-CRP: high-sensitivity C-reactive protein; PaO2: partial pressure of oxygen; FiO2: fraction of inspired oxygen.

	Total	Hospital-acquired pneumonia		P-value
	(n = 139)	With	Without	
		(n = 21, 15%)	(n = 118, 85%)	
Reference values				
Ferritin (n = 138)				0.692
>350	126	20	106	
<350	12	1	11	
Hs-CRP (n = 139)				1
Low risk	1	0	1	
Average risk	0	0	0	
High risk	138	21	117	
D-dimer (n = 92)				0.063
>500	73	13	60	
0 to 500	10	0	19	
Procalcitonin (n = 120)				0.664
Low risk	94	14	80	
Intermediate	22	4	18	
High risk	4	1	3	
PaO2/FiO2 (n = 138)				0.781
>300	34	4	30	
<300	104	16	88	

## Discussion

HAP was diagnosed in 21 (15%) of the patients in the study population. The proportion of patients diagnosed with HAP in our study is similar to previous retrospective studies showing the rate of bacterial coinfection among severe COVID-19 patients who were given tocilizumab. However, none of these studies showed the rate of HAP separately from other bacterial coinfections. A retrospective study done by Campochiaro et al. in 2020 compared severe COVID-19 patients with elevated inflammatory markers who were given either one or two doses of tocilizumab with a patient population that was not given tocilizumab. They found that bacterial or fungal infections were recorded in 13% of patients who were given tocilizumab compared with 12% of patients who were given standard treatment. The rate of bacterial superinfection was significantly lower in patients who were given one dose of tocilizumab (9%) compared with patients treated with two doses of tocilizumab (33%) [18]. A similar retrospective study by Quartuccio et al. showed that 18 out of 111 patients (16.2%) who received tocilizumab developed a bacterial coinfection compared with patients who received a standard of care [23].

However, it is important to note that the standard of care for COVID-19 patients in 2020 was different from the standard of care for COVID-19 patients in our study. In the study done by Campochiaro et al., patients who were not given tocilizumab were given hydroxychloroquine 400 mg daily, lopinavir/ritonavir 400/100 mg twice daily, ceftriaxone 2 g daily, and azithromycin 500 mg daily, along with enoxaparin 4000 UI subcutaneously once a day. These differences in the standard of care given to patients could mean that the findings from this study might not be comparable to our study [18].

There is a significant difference between the mean BMI of patients with and without pneumonia. This is consistent with a meta-analysis done in 2022 that confirmed that obesity, among other factors such as female gender, preexisting pulmonary disease, and older age, is associated with an increased risk of severe disease [24]. There is also a statistically significant difference between the median ferritin, D-dimer, and PaO_2_/FiO_2_ values of patients with and without pneumonia. Previous studies have already shown that elevated ferritin and D-dimer values are associated with more severe disease and poorer outcomes in COVID-19 patients [25,26]. A retrospective study done in 2022 also showed that a PaO_2_/FiO_2_ ratio ≤ 200 is a predictor of in-hospital mortality among COVID-19 patients among other factors such as age ≥ 60 years, chronic obstructive pulmonary disease, leukocytosis, lymphopenia, neutrophilia, and declining renal status (estimated glomerular filtration rate < 90) [27].

Although there appears to be no significant difference between the patient groups in terms of age, sex, and the presence of comorbidities, such as ischemic heart disease, hypertension, diabetes, chronic kidney disease, and asthma, this may be the result of the low sample size in our study.

## Conclusions

This study found that 15% of patients diagnosed with severe to critical COVID-19 pneumonia in 2021 who were given tocilizumab developed HAP during their admission. However, there are significant differences in the standard of care given to COVID-19 patients depending on the year and the hospital where the study was conducted. This could mean that the findings from this study might not be directly comparable to previous studies done in 2020 or 2021. For future studies on this topic, a retrospective case-control study could be done comparing a control group of patients who were not given tocilizumab with a population that received tocilizumab. Such a study would be able to determine the odds ratio of developing HAP among patients who received tocilizumab. Other populations could also be investigated in a similar manner such as vaccinated and unvaccinated COVID-19 patients.

In our study, the diagnosis of HAP was made based on a combination of clinical and radiologic findings associated with HAP along with a positive blood or sputum culture during admission. These results were reviewed by a pulmonologist who acted as a consultant advisor for the study. This could be improved in future studies by having an independent blinded committee review all available clinical, radiologic, and microbiologic data to diagnose HAP. Given that many patients who are admitted with severe or critical COVID-19 pneumonia often have multiple decompensated comorbidities, the decision of giving tocilizumab to manage severe COVID-19 should be weighed against the risk of HAP. Identifying patients who are at a higher risk of developing HAP would also allow clinicians to have a higher index of suspicion for when to start empiric antibiotic treatment for HAP.

## Figures and Tables

**Figure 1:**
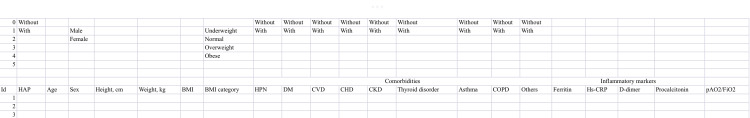
Data collection form HAP: hospital-acquired pneumonia; HPN: hypertension; CVD: cerebrovascular disease; DM: diabetes mellitus; CKD: chronic kidney disease; COPD: chronic obstructive pulmonary disease; Hs-CRP: high-sensitivity C-reactive protein; PaO2: partial pressure of oxygen; FiO2: fraction of inspired oxygen.
